# Emergency Department Admissions During COVID-19: Implications from the 2002–2004 SARS Epidemic

**DOI:** 10.5811/westjem.2020.5.48203

**Published:** 2020-06-23

**Authors:** Muhammad M. Munir, Russell S. Martins, Asad I. Mian

**Affiliations:** * Aga Khan University Hospital, Karachi City, Sindh, Pakistan; † Aga Khan University Hospital, Department of Emergency Medicine, Karachi City, Sindh, Pakistan

## Dear Editor

The emergency department (ED) represents a frontline in the response to the COVID-19 (coronavirus disease 2019) pandemic. This is similar to the 2002–2004 SARS-CoV-1 (severe acute respiratory syndrome coronavirus) epidemic, where EDs played an important role in triage and screening of patients presenting to hospitals. In this letter, we review the impact of the SARS epidemic on hospital ED admissions, and discuss implications for COVID-19 to enable healthcare systems to better anticipate and manage the effects of the current pandemic on the ED.

During the peak of the SARS outbreak, studies from affected countries (predominantly high-income countries such as Taiwan, Singapore, Hong Kong, and Canada) reported overall declining ED visits, especially for high-acuity and non-respiratory emergencies. 1–9 Rates of acute myocardial infarction, pulmonary embolism, and gastrointestinal bleeding presenting to EDs declined, indicating that some seriously ill patients did not get access to appropriate medical care. 1 Possible reasons for declining ED visits included patient fear of contracting SARS from EDs, official announcements deterring ED visits, and the media’s portrayal of the disease. 2, 3, 10 However, some EDs reported an increase in patients harboring concerns of SARS infection, occasionally even without any respiratory symptoms. 6 Symptomless patients posed a challenge to EDs, as overburdened healthcare workers often delayed the full assessment of these patients although they could represent asymptomatic but infective sources of SARS. Moreover, the increase in potential SARS patients visiting EDs deterred not only healthcare-seeking behavior, but also healthcare-providing behavior due to fear of nosocomial transmission and insufficient isolation facilities. 3 However, despite a decline in number of visits, ED staff were increasingly overburdened with the triage and management of the influx of potential SARS patients. 3 As a result, EDs also saw a drop in performance and quality of care indicators, such as length of stay and early return to the ED. 4 Moreover, although expenses in the ED fell, the increased per patient expenditures (up to 35.9%), decreased reimbursements (up to 21.7%), operational disruptions, and decreased surgical procedures placed hospitals under major financial stress. 3, 7 Hospital recovery time, in terms of ED visits, ranged from months to years. 5

Despite most SARS data discussed in this letter originating from high-income countries, we expect the COVID-19 pandemic to produce similar – though perhaps more augmented – effects on ED trends worldwide. The public’s fear of COVID-19 resulting in decreased ED visits for emergencies risks serious health consequences that must not be overlooked. Hospitals must explore ways to reduce these unfortunate consequences, such as the use of telephone helplines encouraging the use of hospital services when appropriate. Telehealth also enables hospitals to continue providing consultations for other medical specialties, thereby reducing financial losses. Reducing the likelihood of a nosocomial COVID-19 outbreak, while also alleviating the public’s fear of visiting an ED, may be achieved through better infection control measures and availability of appropriate personal protective equipment. Where possible, the construction of isolation centers (away from existing EDs), and designation of specific public hospitals for the testing and management of COVID-19 patients, could also offer potential solutions. Additionally, it is also important for public health systems to maintain constant, positive, yet transparent, communication with patients and families through the pandemic. Lastly, decreased revenue from declining visits to EDs may cripple a hospital financially and quickly render it incapable of continuing health provision during the pandemic. To negate this, it is important for governments to mobilize financial resources to compensate hospitals and healthcare workers, ensuring their ability and motivation to continue fighting COVID-19.

In conclusion, the 2002–2004 SARS outbreak showed how the current COVID-19 pandemic may lead to considerable ramifications for emergency care in the population, as well as hospitals’ long-term operational and financial capabilities. Lessons learned from the SARS outbreak show the need for extensive telehealth services, designated COVID-19 management facilities, higher sanitary and infection control standards, and better communication with the general population. This letter aims to guide public health officials to prevent avoidable, yet potentially dire, consequences of the COVID-19 pandemic on ED accessibility and utilization.
